# “I have always lived with the disease in the family”: family adaptation to hereditary cancer-risk

**DOI:** 10.1186/s12875-022-01704-z

**Published:** 2022-04-23

**Authors:** Eliana Silva, Pedro Gomes, Paula M. Matos, Eunice R. Silva, João Silva, Catarina Brandão, Fernando Castro, Maria Carolina Neves, Célia M. D. Sales

**Affiliations:** 1 Center for Psychology, University of Porto (CPUP), Porto, Portugal; 2 Faculty of Psychology and Education Sciences, University of Porto, Rua Alfredo Allen, 4200-135 Porto, Portugal; 3 Cancer Genetics Group, Research Centre of IPO Porto (CI-IPOP) / RISE@CI-IPOP (Health Research Network), Portuguese Oncology Institute of Porto (IPO Porto) / Porto Comprehensive Cancer Centre (Porto.CCC), Porto, Portugal; 4 Psychology Service, IPO Porto Research Centre, Portuguese Oncology Institute of Porto, Porto, Portugal; 5 Department of Genetics, IPO Portuguese Oncology Institute of Porto, Porto, Portugal; 6 Gastroenterology Department, IPO Portuguese Oncology Institute of Porto, Porto, Portugal; 7 Surgical Oncology Department and Breast Clinic, IPO Portuguese Oncology Institute of Porto, Porto, Portugal

**Keywords:** Hereditary cancer syndrome, Patient and public involvement, Family adaptation, Qualitative study

## Abstract

**Background:**

Hereditary cancer syndromes have been conceptualized as a family level process. The present study explores the complexity and challenges of family adaptation to the hereditary cancer syndrome, in the context of genetic counseling and long-term cancer risk management and follow-up surveillance.

**Methods:**

We performed semi-structured interviews with 13 participants with one of the following hereditary cancer syndromes: Lynch Syndrome, Hereditary Diffuse Gastric Cancer Syndrome, Hereditary Breast and Ovarian Cancer Syndrome, or Familial Adenomatous Polyposis. The interview was developed through a participatory approach with the involvement of healthcare professionals and individuals with first-hand experience of living with the hereditary cancer syndromes.

**Results:**

The family is the main source of information and emotional support to deal with hereditary cancer syndromes. Multiple individual adaptation processes and communal coping networks interact, influencing the emotional and health-related behavior of family members. This is affected and affects the family’s communication and its’ members reactions to disclosure, with consequent changes in relationships.

**Conclusions:**

The systemic interdependent dynamics of family adaptation calls for family-centered care of genetic cancer syndromes.

## Background

Approximately 5 to 10% of all cancer cases are caused by inherited pathogenic genes. Carriers of these pathogenic variants suffer from hereditary cancer syndromes, and present an increased risk of developing cancer over the course of their lives [1–3]. The management of these genetic conditions is a family-level process that involves sharing clinical information with family members who may also have inherited the syndrome and need to undergo diagnostic testing [4–7]. In addition, a positive genetic test and decisions about risk-reduction procedures may cause distress not only in pathogenic variant carriers, but also in their family members [8].

The Family System Genetic Illness (FSGI) model provides a framework for understanding how families adapt to genetic conditions, in a dynamic process across four temporal phases [9]. The first phase involves awareness and knowledge about the potential genetic risk. For many families, witnessing cases of cancer or cases of hereditary cancer syndromes provides the reference for which risk is interpreted and integrated into individual life [9–12]. In a pretesting phase, when the individual considers undergoing genetic testing, intense levels of distress may affect the applicant and the decision is influenced by individual and family dimensions [13, 14]. In the testing and post-testing phase applicants cope with their positive or negative results, while integrating this knowledge into their personal and family life [9]. Some individuals talk easily about their genetic testing results with their family, whereas others adopt restricted communication [15–17]. In general, sharing genetic testing results with family members seems to strengthen family relationships and cohesion or led to isolation [18]. Finally, in a long-term adaptation phase, genetic risk is integrated into individual and family identity [9].

Previous research has been studying the systemic impact of a hereditary cancer syndrome. However, most of them are focused on a specific syndrome (e.g., Lynch syndrome) [11, 15], a specific dimension (e.g., family communication; family relationships) [18], and a specific temporal phase in this process of coping with genetic risk (e.g., genetic testing) [7, 18]. Furthermore, it is unclear whether these dimensions of systemic impact are specific to the temporal phases of genetic risk, defined by FSGI, or cross-cutting across this process. Thus, the main goal of this study was to explore the complexity and challenges of family members’ adaptation to the hereditary cancer syndrome, in the context of genetic counseling, long-term cancer risk management, and follow-up surveillance.

## Methods

### Design

We interviewed individuals with hereditary cancer syndromes with a focus on how their risk affected their family and, reciprocally, how their family affected their own individual adjustment. Including individuals with one of the most prevalent and/or studied hereditary cancer syndromes (hereditary breast and ovarian cancer syndrome, HBOC; Lynch Syndrome, LS; familial adenomatous polyposis, FAP; hereditary diffuse gastric cancer syndrome, HDGC), with different risk-management approaches, may help to achieve a more comprehensive picture of the effects of the hereditary cancer syndrome in family life along with the different phases of the process of coping.

### Participants

Participants were recruited from a Portuguese Oncology Hospital. Data collection occurred between July 2019 and February 2020. Potentially eligible participants were identified by two physicians responsible for their risk management process. These physicians were asked to choose participants with different adjustment processes, according to their clinical perception based on several consultations over time, distinct decisions regarding risk management, and to exclude participants who had cancer at the time of genetic testing. Thirty-two individuals met these criteria. Sampling process terminated when no new information arose in the later interviews. Thirteen individuals agreed to participate and gave written informed consent.

Our sample included 13 individuals (6 females, 46.2%), aged 22 to 53 years old (*M* = 35.08; *DP* = 9.81). Most participants were married or living together (*n* = 8; 61.5%), with children (*n* = 7, 53.8%) ranging in age from 3 to 24 years old. Concerning clinical data, mean time since genetic testing was approximately 7 years (*SD* = 5.15; range 2–17 years). Participants were identified with one of the four following hereditary cancer syndromes: HBOC (*n* = 3), LS (*n* = 5), HDGCS (*n* = 4), or FAP (*n* = 1). All participants have already a family member in whom a mutation has been identified (index cases) within their families. Most participants had no previous history of cancer (*n* = 11, 84.6%). Regarding the risk management procedures, most participants opted for prophylactic surgery (e.g., mastectomy for the HBOC syndrome, gastrectomy for the HDGC syndrome, and colectomy for the PAF syndrome; *n* = 9; 69.3%) and some for surveillance according to protocol (e.g., regular colonoscopies for the LS; *n* = 4, 30.8%) (Table 1).

**Table 1:** Participants’ sociodemographic and clinical data

	*n*	*%*
*Sociodemographic characteristics*		
Gender		
Female	6	46.2%
Male	7	53.8%
Mean age (range)	35.08 (22–53)	
Marital status		
Single	3	23.1%
Married/Living together	8	61.5%
Divorced	2	15.4%
Having children	7	53.8%
Children mean age (range)	8.70 (3–24)	
*Clinical data*		
Mean time since genetic testing (range)	7.15 (2–17)	
Hereditary cancer syndromes		
LS	5	38.5%
HDGC	4	30.8%
HBOC	3	23.1%
FAP	1	7.7%
Previous history of cancer	2	15.4%
Risk management approaches		
Prophylactic surgery	9	69.3%
Surveillance according to protocol	4	30.8%

### Procedure

Data were collected at the hospital or at the participants’ home through retrospective semi-structured in-person interviews, lasting, on average, approximately 90 min (range 54–167 min). We developed an interview guide [19] to explore participants’ views on their own adaptation and that of their families, focusing on four critical phases defined by the FSGI model [9]: 1) before genetic testing, 2) during genetic testing, 3) after genetic testing results, and 4) long-term adaptation. Participants were invited to discuss additional information not covered in the questions at the end of the interview.

The study followed a participatory approach [20, 21]. We had the collaboration of six adults (5 females, 83%) with first-hand experience of living with the hereditary cancer syndromes under study for 5 to over 20 years, as well as two clinical oncologists with over 20 years of experience; both groups helped refine the sampling and recruitment design, and gave feedback to the interview guide, in several face-to-face meetings. This participatory approach further contributed to a more accurate identification of sensitive points along with the adaptation to increased risk of developing cancer; the suggestion of interview topics, and a greater understanding of emotional reactivity during the recall process that helped the researchers better prepare for the interviews. Participants in the interviews received a €15 gift card. The study was approved by the Hospital Ethics Committee (Doc. CES-IPOP 04_2017) and the study procedures were performed in accordance with the Declaration of Helsinki.

### Data analysis

**Fig. 1:**
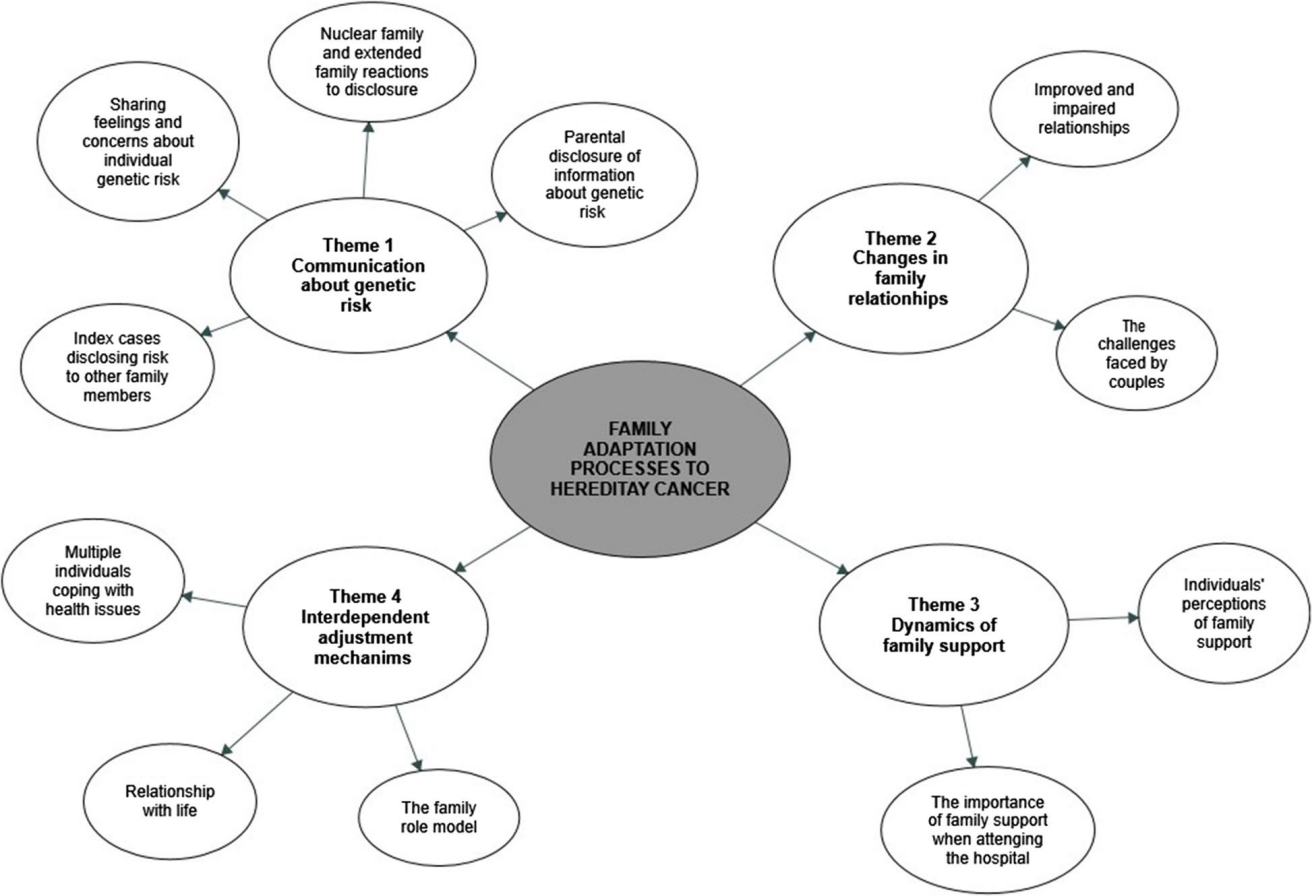
Identification of themes and subthemes concerning family adaptation processes to hereditary cancer

Interviews were audiotaped and transcribed verbatim in an anonymized manner. We selected thematic analysis to identify and report themes and patterns within the data [22]. We chose an inductive approach to data coding and analysis as the predominant trend, informed by a search of relevant literature to better understand the constructs under study. Thematic analysis began with familiarizing us with the data by reading and re-reading the transcripts; identifying the most frequent themes that best represented the participants’ narratives; and reviewing the themes in relation to the coded data and the entire data set, in a recursive process of refinement, also by comparison with the literature and by consensus of the research team. Quotes from participants were included verbatim. A thematic figure (Fig. 1) was developed in QSR’s NVivo software, version 20.2.0.426.

Four main themes and their subthemes emerged (as shown in Fig. 1): communication about genetic risk; changes in family relationships; dynamics of family support; and interdependent adjustment mechanisms.

### Theme 1: communication about genetic risk

This theme included four subthemes: index cases disclosing risk to other family members, sharing feelings and concerns about individual genetic risk, nuclear family and extended family reactions to disclose, and parental disclosure of information about genetic risks to children.

#### a. Index cases disclosing risk to other family members

One participant explained that "I have always lived with the disease in the family, since I was a little girl. I lost my grandfather at the age of three to cancer. That's where the genetic mutation comes from” [2, HBOC]. This quote demonstrates that transmission of information among family members can be widespread when the syndrome affects several members for a long time. The index case passed on genetic risk information to other at-risk family members, even when they were genetically distant relatives, or when they were geographically distant family members. The recognized value of genetic testing, not only for the index case, but also for relatives at risk, led some participants (n = 4) to continuously advise and encourage their family to undergo genetic testing and increase clinical surveillance. Moreover, the importance of the inherited genetic mutation and to acting on this information seems to depend on whether the index case was a first or second-degree relative:

*“She [second cousin] sent us a letter telling us to do the genetic testing […] But we didn’t pay attention, right? We only paid attention later, when the cases began coming up in close relatives” [12, LS].*

#### b. Sharing feelings and concerns about individual genetic risk

Different individuals may be more or less willing to share their experiences regarding testing results and risk management approaches with their families. Some participants (*n* = 5) were opened to sharing their clinical information *“Since my sister and I have the same genetic mutation, we both talk” [12, LS]*. However, most participants described a selective pattern of communication that did not include the whole family*.* For most participants, withholding information was a personal decision to protect themselves from situations of vulnerability *“So, I kept my peace of mind” [4, HDGC]* and the protection of family “*keeping it a little secret because my aunt had passed away and my cousins were still a bit down” [4, HDGC].* Sometimes the decision not to tell the rest of the family was influenced by close relatives:

*"It wasn’t by me. […] but my mother "Oh, you are going to tell, then they're going to be worried. Oh, don't say anything to anyone” [6, HDGC].*

Keeping this information totally secret is not easy, because family members often ask for the genetic testing results. Also, most of the times all affected family members attend the same hospital for their risk management sessions. However, this discomfort in sharing clinical information changed with the ongoing time towards disclosure:

“The more I talked [with the parents, the only people who knew the results of the genetic testing], the worse I felt [before the prophylactic surgery]. But not now. I have no problem talking about it now [in general, with anyone].” [7, HDGC].

#### c. Nuclear family and extended family reactions to disclosure

The initial reactions upon receiving news of a genetic mutation are varied within and between families. Parents’ reactions ranged from “for my mother, everything was fine” [13, LS] to “it’s like everything was falling apart” [12, LS]. The parents’ own experience influenced the adjustment to the testing result, either facilitating it or making it more difficult to accept:

And my mother started crying […] she had not had the surgery yet [prophylactic surgery for the genetic mutation], but she was going to have it in the next few months […] [Her mother said] ‘I am going to go through it [the surgery] and you're going to have to go through it, too’ [5, HDGC].

Some children accepted the family mutation, even when parents seemed to feel guilty for passing the mutation on to them. Among siblings, in some cases (*n* = 2), this topic was discussed *“in a funny way […] and downplaying that problem made me feel relieved and comfortable” [13, LS]*, but they also expressed *“sadness”* over the genetic testing results *[11, LS; 13, LS]* and *“nervousness”* over their siblings’ risk-management approaches *[5, HDGC]*. Two participants felt that their romantic partners *“feared for”* them more than perhaps they feared for themselves *[9, LS; 11, LS]*. One participant complained that her husband *“had a depression”*, and *“I took it badly… because I should be the one getting depressed, not him.” [1, HBOC]*. Some partners seemed to have a good adjustment*,* while others did not.

#### d. Parental disclosure of information about genetic risk

Participants recalled when they first learned about a genetic condition, from their parents, and highlighted the role of their developmental stage in their readiness to accept and act on this information:

*“I didn’t mind it because I was 16, 17 years old. […] you're young, you think you're not going to have any problems in life” [laughs] [13, LS]*

Three participants easily accepted undergoing genetic testing and others considered that their parents omitted information. Participants also described their own experience or concern about informing their children. Some of them advised their children to undergo genetic testing and others acknowledged their children’s autonomy to decide, supporting any decision. One mother described how she plans to tell her six-years old daughter, *"I am planning to tell her when she is about 11, 12 years old" [8, FAP]*. Parents considered themselves in need of *“help”* regarding informing their children about genetic mutation *[2, HBOC]*.

### Theme 2: changes in family relationships

This theme included two subthemes: improved and impaired relationships and the challenges faced by couples.

#### a. Improved and impaired relationships

Two participants did not perceive any change in their family dynamics after receiving genetic testing results. However, for some families the disclosure of genetic testing results had a longer-lasting impact on their relationships. Some participants reported a strengthening of their relationships with their partners and parents. Discovering and living with a familial genetic mutation also contributed to family reconnection and initiating new relationships with distant relatives:

*“She is a second cousin and so we have practically no contact. We had now because of this [both make the same prophylactic surgery]” [5, HDGC].*

On the other hand, some intimate partner relationships weakened, in married couples:

*If I hadn’t had the disease [the hereditary genetic mutation] I would still be married, because I already had children […] He said he didn't want children with me [because of the hereditary genetic mutation]” [8, FAP]; as well as in dating couples “It was a bad reaction […] from my boyfriend's family […] I was with his mother, and she asked me ‘and you still want to have children? […] If it were me, I wouldn't have children’. [...] I ended up ending this relationship, not exactly for my boyfriend, but for his family. [5, HDGC].*

#### b. The challenges faced by couples

Some of the couple’s first thoughts after learning the genetic testing results were about possible implications for their children and family planning. Some couples revealed awareness of reproductive options while others decided not to have children.

Another challenge faced by couples is dealing with prophylactic surgery. Four women with FAP and HBOC syndromes explained the implications of their risk management approaches for couple intimacy “*I had to have sex with my husband with the pouch, which was a horrible thing for me” [8, FAP].* Three participants who underwent prophylactic mastectomy mentioned their experience in terms of body image. While one participant showed her body to her husband *“without any problem” [3, HBOC]*, another participant revealed *“fears” [1, HBOC].* These fears were shared with partners, who seemed to accept and help cope with these changes:

*“He [husband] was very supportive. […] he always handled it very well and helped me to face my demons, right? Like understanding my limitations and being able to digest them as a couple”. [2, HBOC].*

### Theme 3: dynamics of family support

This theme included two subthemes: individuals’ perceptions of family support and the importance of family support when attending the hospital.

#### a. Individuals’ perceptions of family support

All participants considered that *“the biggest support is from family” [12, LS].* It was an important *“help”* to uplift when they heard their family *“telling not to worry” [10, LS],* a source of confidence and emotional *“security” [13, LS]*. Support is *“talking” [10, LS]*, *“being available” [4, HDGC],* and “*giving strength” [10, LS]*. Even in cases where participants did not ask for support, knowing that they can ask for it anytime they need it is reassuring. Participants not only received, but also provided support to their relatives:

*“We are very confident, both me, my father, and my mother, so we support each other. […] I leaned on her, and she leaned on me […] since we are both in the same situation”. [6, HDGC].*

#### b. The importance of family support when attending the hospital

Most participants appreciated having the family company when they attended the hospital in the context of genetic counseling, long-term cancer risk management, and follow-up surveillance:

*Because it’s a hard time for people, right? And that made the environment not so heavy. […] [in general] when people are accompanied, they feel happier, in a better mood, they talk and maybe they forget a little bit about the space [hospital] where they are and what they are doing there. [9, LS]*

This company should be *“a person who loves us" [8, FAP]*. One participant described the suffering she felt when she did not have her husband’s company to receive her genetic testing results:

*I was crying […] I said, ‘come with me to the physician tomorrow’. His [ex-husband] response was straightforward, like ‘you are the one who is sick, I'm not going to miss my job because of an appointment […] you take my mother with you’” [crying]. [8, FAP]*

A small number of participants (*n* = 3) preferred to be alone when attending the hospital. In some cases, even when participants decided to go to the hospital alone, they missed the family company:

*“I was just crying and thinking ‘why did I come alone?’ [receiving the result of the genetic testing] I have no doubts that it would have helped to have someone with me at that time” [2, HDGC].*

### Theme 4: interdependent adjustment mechanisms

This theme included three subthemes: the family role model, relationship with life, and multiple individuals coping with health issues.

#### a. The family role model

Family cancer history plays a significant role in coping with cancer risk. Individuals often relive painful family members’ past experiences, especially when attending the same hospital:

*“As soon as I walked into that gate, I immediately remembered her [aunt who died] […] it was a bit difficult” [4, HDGC].*

On the other hand, participants are acquainted with the symptoms and treatments associated with hereditary cancer syndrome, from seeing it happen with their relatives, and from talking about it with those who have been through it: *“He [father] was the first close experience I had […] so he was my example” [4, HDGC]*.

The family example seems to have different effects on the individuals. For some of them, it reassured; it gave a preparation for their own case; conveyed emotional security; provided familiarization with the hospital, and experience-based advice. In contrast, some participants reported more negative feelings from knowing the negative experience of family members, such as fear, anxiety, and discouragement. Participants also reported negative thoughts, as well as difficulties in the decision-making process *“There were many things that complicated my decision [about to have prophylactic surgery], like my aunt’s surgery” [7, HDGC].* Individuals also act as a role model to their close relatives *“If I reacted badly, she [sister] would probably react the same way […] She sees me as a mirror” [7, HDGC]*.

#### b. Relationship with life

Witnessing the experiences of family members and particularly the cases in which their relatives die of cancer raised the *“fear of dying young” [1, HBOC]* and a reduced life expectancy. In general, participants shared how they changed their perspectives about their life and their future.

*It just changed my way of thinking, of not thinking about the future. [...] enjoying life to the fullest. Since then, I don’t work on Saturdays. […] I don't look at it like 'I have to set everything up for the future', no I don’t. [...] Something tells me that my future isn’t going to be very long. [12, LS]*

#### c. Multiple individuals coping with health issues

Individuals with an inherited genetic mutation have to cope with their health condition and that of their affected relatives, who sometimes are living the same process*.* In some cases, this experience can be very difficult, as illustrated by the following quote:

*He [uncle] got sick after I had surgery and was in recovery. [...] he is like a brother to me [...] There were times when ideas passed through my mind to leave this world. [...] I couldn’t stand to see so much suffering anymore, not from the family, right? [...] It was like an explosive grenade. [10, LS]*

Mutation carriers also see their adjustment affected by other emerging problems in the family. Sometimes the concomitant sources of family distress resulted in postponing genetic testing “*Because I was also very busy with my son's things and work stuff” [1, HBOC],* or postponing health care *"My mother had surgery, came home […] I had to be my mother's mother […] Yes, my own health came last. [2, HDGC]*.

## Discussion

This study is one of the few to explore the impact of hereditary cancer syndromes on the family system throughout the different phases of family adaptation to a genetic condition. A central insight emerging from analysis was the complexity of multiple individual adaptation processes taking place in a family suffering from a hereditary cancer syndrome. Current experiences bring back memories of prior family experiences, which may influence individuals’ adjustment and health decision-making, depending on the relatives’ reactions and the success of their risk reduction approaches. The role of family health history as the primary basis that individuals use to formulate their perceptions of cancer risk has been emphasized by others [12]. Also a previous study [18] found that the index case acts as a role model for other relatives. However, narratives of participants in our study suggest that this pressure to act as a role model can be present in any person within the family. In other words, our data brings out interdependent dynamics of role modeling, where a person learns from previous or ongoing experiences of other members with the disease; while simultaneously feels pressured to act as a role model for other close relatives. Interviews revealed networks of family support dealing hereditary cancer syndrome as a shared stressor. These mechanisms of communal coping (collaborative processes to deal with a distressing event that is perceived as a common threat) have been previously identified in hereditary cancer syndromes [4].

In the participants’ perception, family is the primary source of experience-based advice and emotional support over time, namely for advice on how to disclosure testing results, for sharing experiences and advice on risk-reduction procedures, and also to share feelings and concerns. The reported importance of family support can be explained by the activation of individuals’ attachment system [23], manifested in the search for emotional closeness and the need for the physical presence of relatives in the hospital. Some participants illustrated internalized representations of security by saying that what is most important is their perception of availability of family, even when they prefer to be alone. However, although there is a consensus regarding family as the privileged source of support, interviews also revealed a need for individual privacy, which is not always easily accepted by the family. Most participants adopted a selective communication pattern with close relatives, motivated by a sense of personal protection, or protection of the family from distress.

Interviews also brought to light that mutation carriers may face different personal and relational challenges, depending on the life cycle and family context, as posited the FSGI model [9]: For young people, the difficulties and discrimination faced in dating relationships, given the possibility of passing the genetic mutation to future children; young couples’ decision-making about their family planning and available reproductive options; and parents’ perceptions and feelings associated with the disclosure of information about genetic risk to their children. At the same time, young and adult family members are often caregivers of their relatives, and this responsibility can be a source of great pressure and led some individuals to pay less attention to their own health and in some cases even to have suicidal thoughts.

Finally, interviews about family adaptation processes presented several stories of illness experiences. Some participants used warlike metaphors to represent their family lives, similar to that expressed by individuals with fear of cancer recurrence [24]. This portrays a profound sense of vulnerability and emotional suffering when receiving a hereditary cancer syndrome diagnosis. Some participants expressed a reduced life expectancy, and consequently a change in priorities, and the pursuing of increased quality of life. The change in meanings across different personal domains as a result of a perceived existential threat, also called post-traumatic growth, is a phenomenon identified in cancer patients [25], which our study suggests also applies to individuals with hereditary cancer syndromes.

 Some limitations of this study are noted. Our sample included four hereditary cancer syndromes with different risk-management approaches, and thus with different experiences.

Another limitation was the inclusion of only one family member’s perspective on the processes of family adaptation to hereditary cancer. In addition, many of our questions were retrospective, and in some cases, participants were asked to recall events, family reactions, and adaptation processes that had happened several years ago, possibly resulting in recall bias. Further qualitative research should give voice to other family members for a better understanding of the complexity of family adaptation, using prospective designs.

### Practice implications

This study provides insights into some dimensions for improving aspects of care. First, prior clinical experiences of close relatives suffering from cancer or cancer genetic syndromes seems to be an important learning source for mutation carriers. At the same time, these modeling processes may influence decision-making about an individual’s risk-management plan. Therefore, it is advisable that genetic counseling and risk management services screen cancer-related family history and possible (mis)conceptions and fears. At the same time, health literacy contents can be targeted to help challenge perceptions of intergenerational repetition of clinical experiences.

Second, the evaluation of the risks of adaptation to genetic cancer risk can benefit from screening family life-cycle dimensions, such as active diseases in the family, or other stressful live-events that limit the individuals’ attention to their own health care.

Third, family-based interventions for the promotion of psychological adaptation seem to be of particular relevance. For instance, multi-family group interventions [26, 27] have been proven to be cost-effective approaches to provide a safe interpersonal context for this sharing of individuals’ concerns, experiences, and role models, stimulating existing sharing processes for mutual support, while respecting individual privacy; helping the couple deal with family planning and adaptation to prophylactic surgeries, and the disclosure process to their children, among others.

Finally, our results call for a family-centered care approach, based on an interprofessional collaborative teamwork (e.g., geneticists, surgical technicians, physicians, psychologists) that discusses and monitors family cases. Generally, several family members attend the same hospital, but the individual-centered approach does not allow healthcare professionals to capture the family-level health related dynamics found in this study.

## Data Availability

The datasets generated and/or analysed during the current study are not publicly available due [to data protection of participants] but are available from the corresponding author on reasonable request.

## Abbreviations

FSGI: Family System Genetic Illness model
HBOC: Hereditary breast and ovarian cancer syndrome
LS: Lynch Syndrome
FAP: Familial adenomatous polyposis
HDGC: Hereditary diffuse gastric cancer syndrome
